# Surgical treatment of proximal humeral fractures with the transosseous suture fixation

**DOI:** 10.1186/s13018-021-02555-7

**Published:** 2021-06-23

**Authors:** J. Miquel, R. Martínez, F. Santana, P. Marimon, C. Torrens

**Affiliations:** 1 Corporació Sanitària Parc Taulí, Parc Taulí, 1., 08208 Barcelona, Spain; 2 Consorci Sanitari de l’Anoia, Avda Catalunya, 11, 08700 Igualada, Spain; 3 Parc de Salut Mar, Passeig Marítim, 25-29, 08003 Barcelona, Spain

**Keywords:** Proximal humeral fractures, Transosseous fixation, Open reduction and internal fixation

## Abstract

**Background:**

The surgical treatment of displaced proximal humeral fractures is commonly affected by implant-related complications. We evaluated the functional and radiographic results of transosseous suture fixation in a series of displaced proximal humeral fractures (PHF).

**Methods:**

Sixty-four patients were retrospectively classified by age, sex, and the Neer fracture classification. Two-part greater tuberosity (2pGT) displaced fractures and 3-part (varus and valgus) and 4-part valgus impacted fractures were managed with fragment reduction and transosseous suture fixation. Patients with minimum follow-up of 24 months and assessed with the Constant-Murley score (CMS) were included. Radiological and medical complications, as well as reinterventions were also recorded.

**Results:**

Forty-six patients with a mean follow-up of 58 (24–132) and a mean age of 58 years old were analyzed. Patients with 2pGT (*n* = 10) fractures had a CMS of 76 points (59–89); patients with 3-part fractures (*n* = 22) had a score of 67 points (13–91); and those with 4-part fractures (*n* = 14) had a score of 64 (24–76) points. The overall complication rate was 6 out of 46, and 4 patients required reintervention for different reasons. Patients presenting with 3-part varus fractures had significantly lower functional outcomes scores (*p* = 0.007). Humeral head osteonecrosis was present in 9 patients and significantly affected the functional outcomes (*p* < 0.05). However, only three out of nine patients with osteonecrosis required subsequent surgery at the indicated follow-up.

**Conclusions:**

The fracture reduction and transosseous fixation technique represents a safe technique with low complication and reintervention rates. The presence of humeral head necrosis did not lead to subsequent surgical intervention because no hardware had protruded.

**Level of evidence:**

Level IV, retrospective study

## Background

The increase in surgical treatment for displaced proximal humeral fractures (PHF) is associated with the evolution of medical device availability for osteosynthesis. Several surgical treatment modalities have been developed over the years, such as the use of different plate designs, nails, and percutaneous techniques. The complications reported are related to the design of the implants [1–3], and the complication rate can be up to 40% [4]. Encouraging reports were published when locking plates were first available for proximal humeral fractures [5]. However, long-term follow-up demonstrated a high complication rate [4], while the loss of reduction and subsequent cut-out were the most prevalent problems related to these implants [6]. In the event of humeral head avascular necrosis (HHAVN) or secondary varus collapse, the fixation provided by head screws may potentially cause hardware-related complications with subsequent articular damage. The use of humeral nails for complex PHF may be considered a challenging procedure, as functional outcomes and complications are strongly influenced by the grade of achieved reduction [7].

Jacob et al. [8] described the four-part valgus impacted fracture pattern, introducing the concept of anatomic restoration by ascending the impacted humeral head to permit reduction of the tuberosities. Since then, different authors have reported a similar approach with different fixation techniques [9–11]. More recently, least fixation techniques have been described by using transosseous sutures to stabilize four-part fractures, with or without disimpaction of the articular head fragment from its valgus position [12, 13]. However, limited data are available regarding complex PHF treated with anatomical restoration and bony fragment stabilization with the use of transosseous sutures.

The present study aims to describe (1) the clinical outcomes of patients with displaced proximal humeral fractures treated with transosseous sutures without hardware implantation and (2) the radiographical outcomes of patients treated with the mentioned technique.

## Materials and methods

### Study design and patient selection

This was a retrospective study recruiting patients treated with the open reduction and osteosuture fixation technique for acute, displaced proximal humeral fractures from 2001 to 2018 in two different hospitals. Three different surgeons performed the surgical treatment (JM, FS, and CT). The indications for treating patients with the mentioned technique were (1) an isolated, displaced two-part fracture of the greater tuberosity (2pGT) with or without anterior dislocation of the humeral head, (2) a three-part fracture, or (3) a four-part valgus impacted fracture. Patients presenting with two-part fractures of the surgical neck and humeral head split fractures were not considered for the osteosuture technique.

The inclusion criteria for the purpose of this study were (1) patients presenting with an indication for the osteosuture technique and (2) patients assessed within the first 3 weeks after the injury occurred. Patients unable to reach a minimum of 24 months of follow-up were also excluded from the study.

### Surgical technique and postoperative care

The patients were intubated and placed in the beach chair position with waist flexion of 45° to 60° and were draped under sterile conditions. A deltopectoral approach was performed; the cephalic vein was identified and retracted laterally. The deltopectoral interval was developed, and the humeral shaft was identified. The clavipectoral fascia was excised, preserving the coracoacromial ligament. Soft-tissue tenodesis of the long head of the biceps to the pectoralis major tendon was performed with #1 Vicryl (Ethicon, Edinburgh, UK).

#### Fixation of 2-part greater tuberosity (2pGT) fractures

The greater tuberosity (GT) was identified posteriorly to the original native site and tagged with the use of #5 Ethibond (Ethicon, Edinburgh, UK) to allow mobilization. The fracture bed was also identified and cleared of hematoma or initial scar tissue. Three #5 Ethibond sutures were passed behind the GT or through the bone, in the upper, middle, and lower portion of the tuberosity fragment, as previously described by Dimakopoulos et al. [13]. The remaining tails of the sutures were passed from the fracture bed to the lesser tuberosity with the use of a Mayo needle (Mckesson Medical-Surgical Inc., TX, USA). The GT was reduced to its original site with the use of mobilization sutures and fixed by tying the sutures. Intraoperative fluoroscopy was used to assess the quality of the reduction when required by the surgical team.

#### Three-part valgus and 4-part valgus impacted fractures

The same approach was used for the 3-part and 4-part valgus impacted fractures. The fracture lines between the tuberosities were identified and gently separated. This maneuver allowed access to the humeral head, which was found to be valgus impacted in the humeral shaft. The humeral head was gently disimpacted to correct the valgus position in order to allow proper reduction of the greater and lesser tuberosities. The tuberosity or tuberosities were reduced adequately in the axial plane. Three horizontal #5 Ethibond sutures were passed from the GT to the lesser tuberosity (LT), providing stable fixation of the construct. Intraoperative fluoroscopy was used to assess the quality of the reduction when required by the surgical team**.**

#### Three-part neutral and varus fractures

**Fig. 1:**
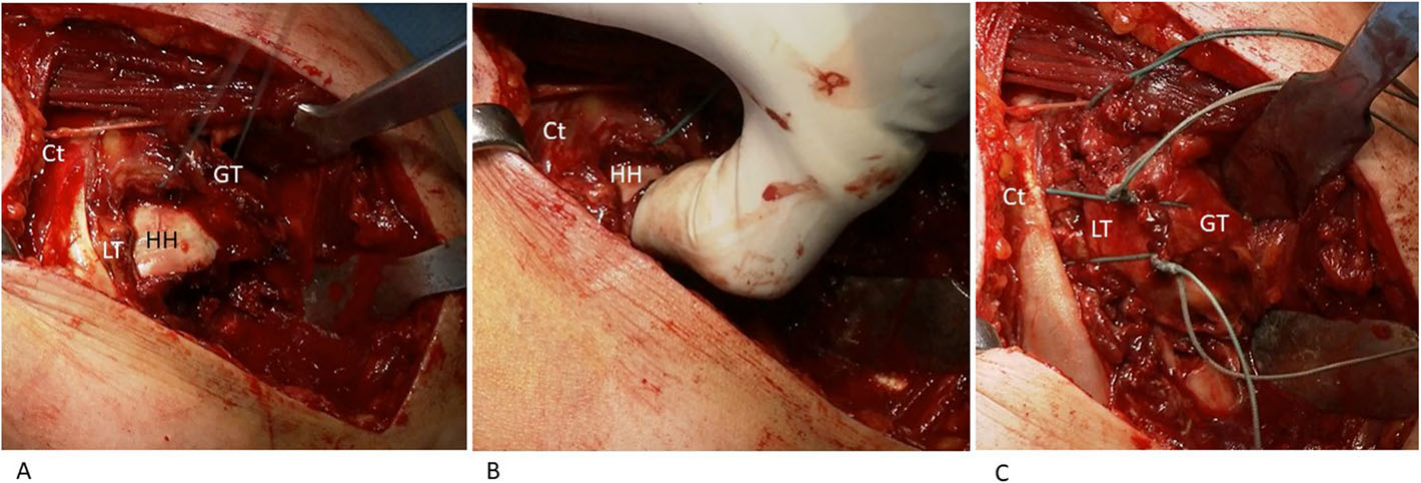
Intraoperative presentation of a 4-part valgus fracture in a 56-year-old female. **A** Humeral head was found impacted on the humeral diaphysis. Greater tuberosity (GT) and lesser tuberosity were found posteriorly and anteriorly to the humeral head. The conjoint tendon (Ct) is also identified. **B** The reduction maneuver disimpacting the humeral head recreates the anatomical space for the tuberosities. **C** Once the reduction is achieved, the construct is stabilized by the use of horizontal sutures

The same principles for the 3-part valgus fractures were used to reduce 3-part neutral or varus fractures. Attention was focused on reducing the GT fragment to the fracture bed site in the proximal humerus. Three horizontal #5 Ethibond sutures were passed from the GT to the LT, providing stable fixation of the construct (Fig. 1). In the 3-part varus fracture scenario, a figure-eight suture pattern was performed with the #5 Ethibond sutures to reduce the humeral head to the humeral shaft.

#### Postoperative care

Patients were immobilized with a regular sling with internal rotation for 3 weeks. The rehabilitation protocol was focused on passive range of motion recovery for the first 6 weeks after immobilization, followed by active-assisted exercises for 3 weeks, and strength recovery for 3 additional weeks. The protocol was supervised by a physiotherapist. Patients were monitored at 3, 6, 12, and 24 months after the surgery. From that point on, the patients were visited annually by the treating surgeon. This protocol was the same for the two hospitals participating in the present study.

From this time forward, patients initiated passive range of motion with the use of a pulley. Strengthening was allowed starting 3 months postoperatively.

## Methods of assessment

The research protocol included the following items: (1) demographic information, including age and sex, (2) the Neer classification, determined by X-rays in the anteroposterior and outlet views [14], and (3) the Constant-Murley score (CMS) used to assess the functional outcomes 2 years after the injury. The mobility items included in the CMS were assessed by using standard goniometers, while strength items were evaluated by using digital dynamometers (IsoForceControl® EVO2, MDS, Oberburg, Switzerland and Lafayette Manual Muscle Testing System, Lafayette Instrument®, Lafayette, IN, USA). Standard follow-up was performed according to the particular protocol for the different centers participating in this study. All patients were invited to complete a 2-year follow-up. The functional analysis was performed at the latest available follow-up visit, with a minimum follow-up of 24 months to detect any potential complications such as humeral head avascular necrosis.

The presence of radiological complications such as avascular necrosis of the humeral head and prospective signs of shoulder joint osteoarthritis was assessed with the use of standard AP and outlet X-ray views of the shoulder at the 2-year follow-up and annually thereafter. Posttraumatic osteoarthritis was defined according to the Kellgren-Lawrence criteria [15]. Humeral head avascular necrosis was defined by the loss of humeral head contour and destruction of the trabecular architecture in an articular segment on AP views following the Cruess criteria [16]. The cephalo-diaphyseal angle was also recorded from the X-rays. Complications and reinterventions were recorded as morbidity outcomes.

The study was submitted to the Institutional Review Board and received approval (IRB number 2012/4815/I).

## Statistical analysis

The continuous variables are presented as the means and ranges. The categorical variables are presented as the number of cases and percentages. Kruskall-Wallis test was used to study the correlation between values of the CMS and categorical values, such as the presence of humeral head avascular necrosis and to compare the values of CMS within the different patterns of 3-part factures (valgus/varus). The level of significance was set at *p* values < 0.05.

## Results

Sixty-four patients met the indications for the osteosuture technique and were operated on within 3 weeks after the injury. Of those, 3 patients did not reach a minimum follow-up of 24 months, and the remaining 15 patients were lost to follow-up. In consequence, 46 patients were able to reach a minimum of 24 months of follow-up and were finally included in the analysis. The mean age was 58 years old (28–86). The sample was composed of 30 females and 16 males. The mean follow-up was 58 months (24–132). Patients underwent operations 11 (1–22) days after the injury.

**Fig. 2:**
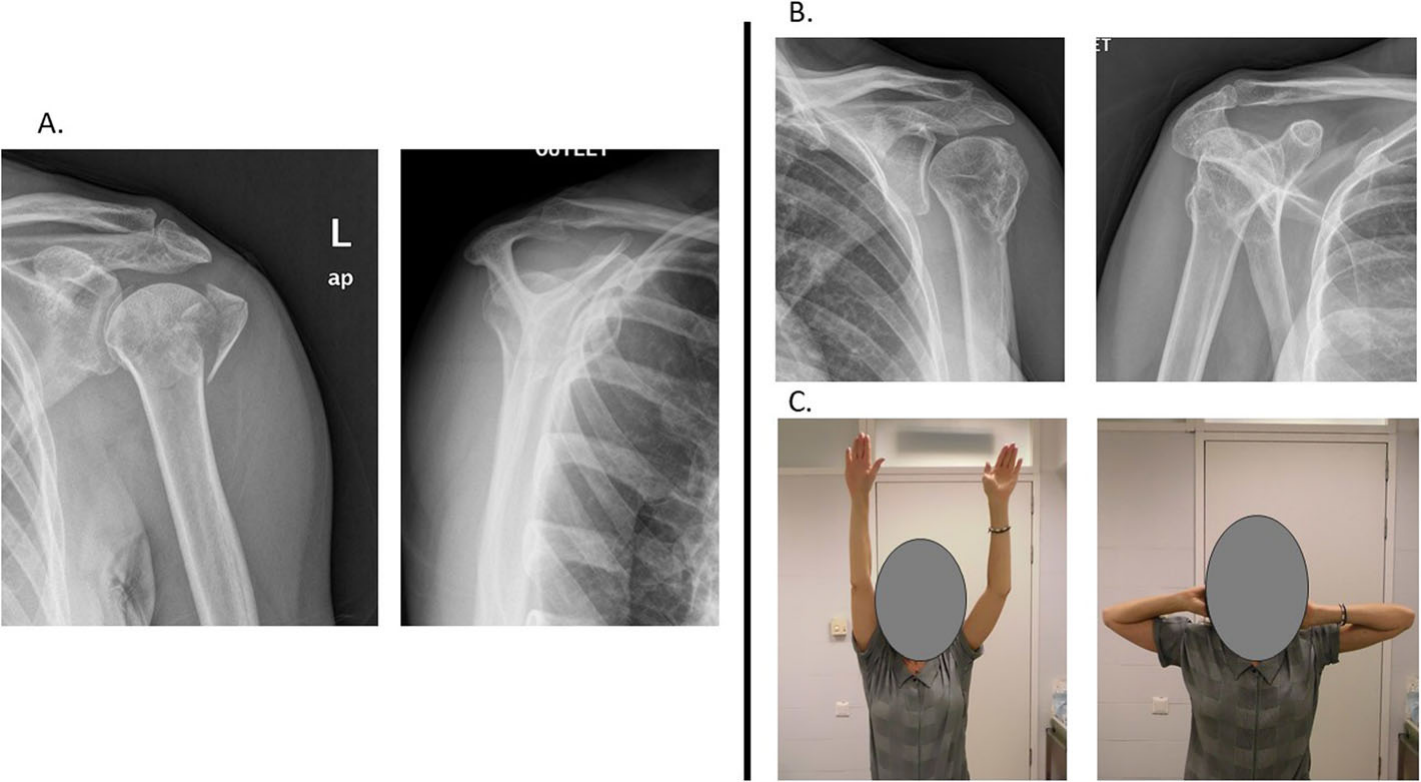
**A** Initial X-ray presentation of a 47-year-old. Female with a proximal humeral fracture. **B** Radiological results achieved at the 2-year follow-up. **C** Clinical results at the 2-year follow-up with a Constant-Murley score of 75 points

The fracture pattern according to Neer classification as well as the functional outcomes recorded with the use of the Constant-Murley are shown on Table 1. Regarding the 3-part fractures, the average Constant-Murley score was 67.15 points. Once the 3-part fractures were subcategorized according to the initial injury pattern, the 3-part valgus impacted fractures obtained scores of 72.26 points, while the 3-part varus or neutral fractures obtained significantly worse outcomes (average 42.52 points (13–72.7), *p* = 0.0013). The 4-part fractures obtained scores of 51.75 points at the average follow-up (Fig. 2).

**Table 1:** The functional and radiological outcomes of patients at minimum 2-year follow-up, measured by the Constant-Murley score

	2-Part GT fractures	3-Part fractures	4-Part fractures
N	10	22	14
Mean age	51.50 (30–70)	62.27 (28–77)	56.15 (33–78)
Pain	12.55	12.63	8.7
Daily life activities	18.66	16.22	13.85
Forward elevation	8.61	7.50	6.85
Abduction	8.20	7	6
External rotation	8	6.45	5.71
Internal rotation	8.40	7.36	6
Strength	11.65	9.64	4.61
Total constant score	75.96 (54–92)	67.15 (13–91)	51.75 (24–76)
Forward elevation (°)	153°	132°	127°
Abduction (°)	144°	120°	110°
HHAVN	0	2	7
Head–shaft angle	Not applicable	130°	128°
Osteoarthritis	1	2	4

*GT* greater tuberosity, *HHAVN* humeral head avascular necrosis

(°) degrees

Concerning the radiological outcomes, a total of 9 patients presented with humeral head avascular necrosis at the indicated follow-up. A total of 7 patients presented with signs of osteoarthritis on X-rays (Table 1).

### Complications and reinterventions

Six patients presented with postoperative complications. Three of them suffered from transient axillary nerve injuries that healed uneventfully with conservative treatment. One patient presented with transient ulnar nerve neuropraxia that also healed with a nonoperative approach. Two patients presented with stiff shoulders postoperatively. This symptom was determined by the limitation of both active and passive restriction of range of motion at a minimum of 6 months postoperatively. Of those, one patient required arthroscopic capsular release 17 months after open reduction and fixation for a 4-part valgus fracture, while the other patient recovered the range of motion with conservative treatment. One patient died due to postoperative bronchoaspiration.

Four patients required reinterventions. The mean time of reintervention was 23.5 (17–36) months after the index surgery. One patient required arthroscopic capsular release for a postoperative stiff shoulder, while three patients required salvage procedures for humeral head avascular necrosis. Hemiarthroplasty was the choice for two of the patients presenting with symptomatic fracture sequelae from 2001 to 2011. The remaining patient was treated with reverse shoulder arthroplasty in 2014 as a salvage procedure. The detailed characteristics of the reinterventions are shown in Table 2.

**Table 2:** Detailed characteristics of the patients required a subsequent surgery after the transosseous suture fixation technique

Patient ID	Gender	Fracture type	Age at the index surgery	Time to reintervention (months)	Reason for subsequent surgery	Procedure
#11	Female	3-part varus	63	22	HHAVN	Reverse total shoulder arthroplasty
#21	Male	4-part	53	36	HHAVN	Hemiarthroplasty
#48	Female	4-part	33	17	Frozen shoulder	Arthroscopic capsular release
#49	Female	4-part	58	19	HHAVN	Hemiarthroplasty

*HHAVN* humeral head avascular necrosis, *ID* identification

**Fig. 3:**
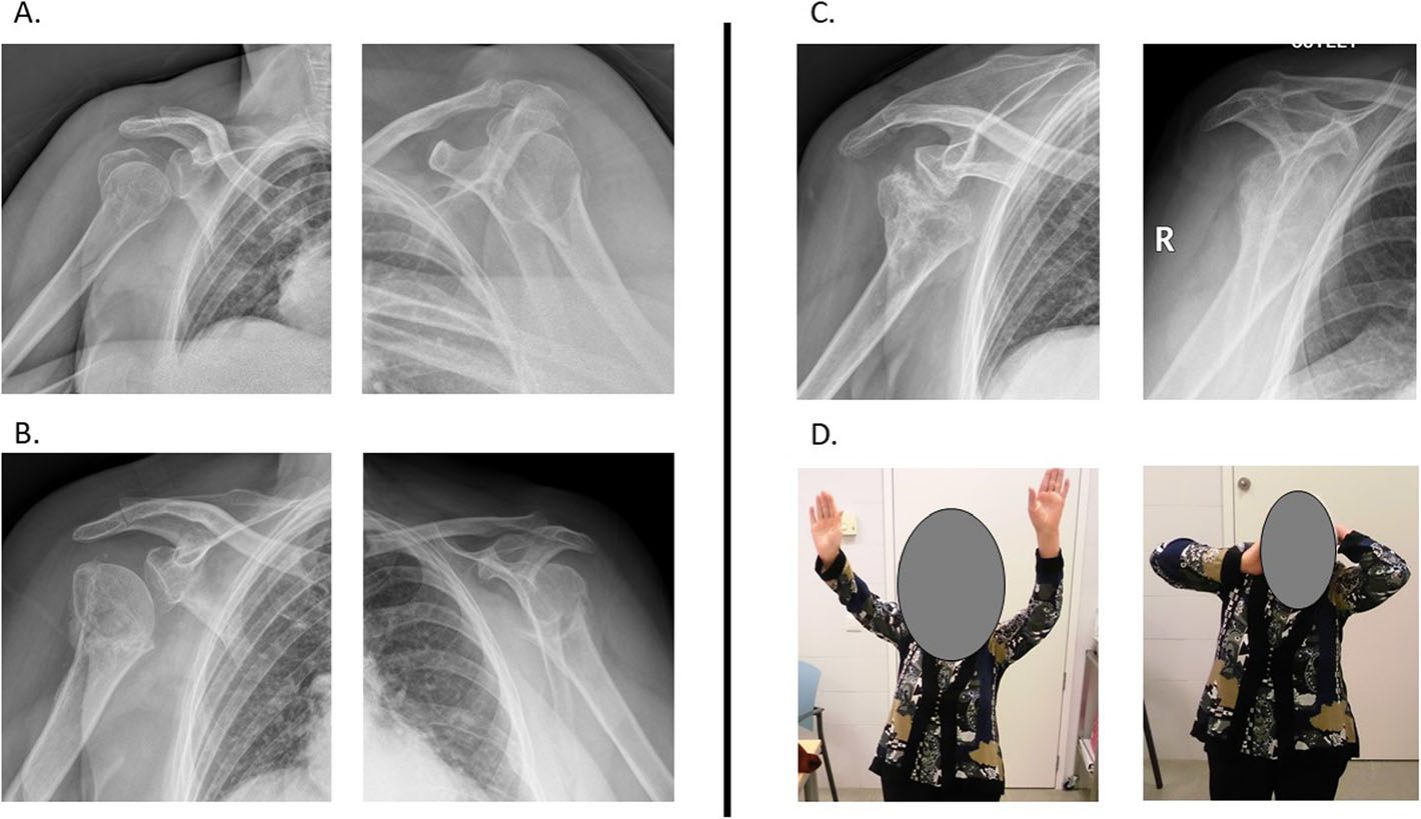
**A** Initial X-ray presentation of a 61-year-old female with a proximal humeral fracture. **B** Radiological results at the 6-month follow-up. **C** Radiological results at the 3-year follow-up. **D** Clinical results at the 3-year follow-up with a Constant-Murley score of 49 points

The presence of humeral head avascular necrosis (HHAVN) impacted the functional outcomes. Patients presenting with HHAVN had CMS of 35.9 points, while patients with preservation of an intact humeral head had average CMS scores of 70.7 points (*p* < 0.01). The presence of HHAVN did not automatically lead to reinterventions in the patients. Three out of nine patients presenting with HHAVN required a salvage procedure. The remaining six patients declined a subsequent surgery, even if it was offered. The mean Constant-Murley score for patients who declined surgery for HHAVN was 41.72 (24–54.30) (Fig. 3). No patients required a salvage procedure for osteoarthritis at the indicated follow-up.

## Discussion

The data presented in this paper support the use of a hardware-free technique to restore bone fragment anatomy in displaced proximal humeral fractures when open reduction and internal fixation is the treatment of choice. Notably, 2-part GT fractures and 3-part valgus fractures demonstrated good Constant-Murley scores at the indicated follow-up. Even though we did not observe any strict technique-related complications, we observed complications in 6 out of 46 patients. The presence of humeral head avascular necrosis did not automatically lead to reinterventions in the patients. Consequently, this technique was associated with a low reintervention rate.

The functional outcomes observed in our series are comparable with those of other series reported in the literature when rigid hardware fixation is implanted in patients with proximal humeral fractures [13, 17]. Patients presenting with 2-part fractures obtained on average good results based on the CMS, with no complications or need for reinterventions. Regarding patients presenting with 3-part fractures, the presence of varus deformity on the original X-ray impacted the final functional outcome. A significant difference regarding functional outcome was found comparing 3-part varus fractures to 3-part valgus or neutral fractures. Hardeman et al. also found [18] that preoperative varus displacement affected postoperative outcomes while increasing the risk of failure when using rigid hardware fixation. Fractures demonstrating varus malalignment are more likely to disrupt the medial soft tissue sleeve and are more likely to be unstable with different fixation techniques. Varus malalignment is a strong predictor of more unsatisfactory outcomes and loss of fixation techniques [19]. As a consequence, we do not recommend the osteosuture technique for patients presenting with 3-part varus impacted fractures. Patients presenting with 4-part fractures obtained fair outcomes according to the CMS, while 3 out of 14 patients with this fracture pattern required subsequent surgery. As we did not use age- or sex-adjusted CMS in our population, the potential positive effect of interventions in this group is likely to be underestimated. However, we were not able to reproduce the results obtained by some other authors [12, 13] using similar techniques for 4-part valgus fractures. As a consequence, we can only recommend the use of the osteosuture technique for patients presenting with 4-part valgus impacted fractures when open reduction and internal fixation is the surgeon's choice.

An increasing number of studies have brought into question the benefits of open reduction and internal fixation with the use of locking plates [20, 21]. Implant-related complications following this technique have been variably reported (0 to 30%) [4, 5]. Beeres et al. [22] reported a rate of implant-related complications of 28%, representing 40% of the total complications in their series. As a consequence, some authors advocate for early hardware removal to avoid implant-related complications [23]. Therefore, high chances of reoperations must be expected when implanting a locking plate for proximal humeral fractures. However, the aim of the present study did not compare the osteosuture technique with a fixation technique using metal hardware.

Regarding the use of nails, symptoms related to rotator cuff disease are the main reported complication, varying from 24 to 73%, depending on the characteristics of the reported series [24, 25]. The revision rate for patients treated with a proximal humeral nail varies from 10 to 42% depending on the nail-specific design, while 20% of revisions with humeral nails are strictly related to the implant [24]. Some authors recommend regular clinical and radiographic follow-up for at least 5 years to detect impending screw perforation and to plan for timely screw removal [17].

The presence of HHAVN was observed in 9 of our patients. Most of the patients presenting with this complication were classified as having 4-part valgus impacted fractures (7 out of 9). The presence of humeral head avascular necrosis impacted the functional outcome when assessed with the Constant-Murley score in our series. However, as a benefit of the hardware-free technique, the presence of avascular necrosis of the humeral head did not subsequently lead to reoperations in the patients. Only 3 out of 9 patients presenting with this complication required additional surgery. The incidence observed in our population was even higher than previously reported experience with the osteosuture technique [26]. The patients' demographics, fracture patterns, and disimpaction of the humeral head in our surgical techniques may eventually explain the disparity in this complication rate.

This study presents several potential weaknesses. First, the sample was heterogenic, as the age ranged from 30 to 85 years old. This may affect the applicability of the study. Second, this was a retrospective study. As a consequence, the hypothesis could not be thoroughly tested given the design of the study.

Different clinical contributions may be adopted from this study. The osteosuture technique represents an option for open reduction and internal fixation with similar clinical outcomes and lower complication/reoperation rates compared with hardware fixation techniques. We recommend the technique for patients presenting with 2pGT fractures, 3-part valgus fractures, or 4-part fractures in young biological patients. Second, the presence of HHAVN does not imply reintervention.

## Conclusions

The osteosuture technique represents a safe fixation technique when considering open reduction and internal fixation in patients presenting with PHF. The complication and reintervention rates were low, and the presence of humeral head avascular necrosis did not automatically lead to reintervention in the patients.

## Data Availability

The datasets used and/or analyzed during the current study are available from the corresponding author on reasonable request

## Abbreviations

2pGT: Two-part greater tuberosity
CMS: Constant-Murley score
Ct: Conjoint tendon
GT: Greater tuberosity
HH: Humeral head
HHAVN: Humeral head avascular necrosis
LT: Lesser tuberosity
PHF: Proximal humeral fractures
